# Discovering the diversity of *Acarosporaceae* with pruina in China

**DOI:** 10.3897/mycokeys.137.201158

**Published:** 2026-07-21

**Authors:** Fuhui Liang, Jiaxin Wang, Ling Hu, Congcong Miao, Hongli Si, Runlei Chang, Lulu Zhang, Xinyu Wang, Lisong Wang, Zuntian Zhao

**Affiliations:** 1 College of Geography and Environment, Shandong Normal University, Jinan 250300, China College of Geography and Environment, Shandong Normal University Jinan China; 2 College of Life Sciences, Shandong Normal University, Jinan 250300, China College of Life Sciences, Shandong Normal University Jinan China; 3 State Key Laboratory of Phytochemistry and Natural Medicines, Kunming Institute of Botany, Chinese Academy of Sciences, 650201 Kunming, China State Key Laboratory of Phytochemistry and Natural Medicines, Kunming Institute of Botany, Chinese Academy of Sciences Kunming China; 4 Yunnan Key Laboratory for Fungal Diversity and Green Development, Kunming Institute of Botany, Chinese Academy of Sciences, 650201 Kunming, China Yunnan Key Laboratory for Fungal Diversity and Green Development, Kunming Institute of Botany, Chinese Academy of Sciences Kunming China

**Keywords:** Lichenized fungi, new record, new species, phylogeny, taxonomy

## Abstract

During our research on *Acarosporaceae* species, we found three new pruinose species, *Sarcogyne bayingolinensis*, *S. parasitica*, and *S. yiliensis*, and one new species to China, *Acarospora iqbalii*, based on a combination of morphological, chemical, and phylogenetic analysis. All three new species have IKI+ blue hymenial gel. *Sarcogyne bayingolinensis* is characterized by lobate thallus margin and no pruina surrounding the apothecial disc. *Sarcogyne parasitica* is characterized by areoles with deep fissures surrounding the apothecial disc and exhibits a parasitic lifestyle. *Sarcogyne yiliensis* is characterized by thin hymenium (50–60 μm), and tall subhymenium (50–80 μm). In addition, we discovered that *A. tianshanica* exhibits two chemical types (with or without gyrophoric acid), and we supplemented the molecular data of *A. glaucocarpa* from China. A key for the *Acarosporaceae* species with pruina reported in China is also provided.

## Introduction

White pruina is an important diagnostic feature for identifying lichen species, particularly within *Acarosporaceae*. Its main component is calcium oxalate, which gives the lichen a frosty, white appearance (Wadsten and Moberg 1985). Moreover, the white surface reflects sunlight like snow, thereby absorbing less heat, while it also retains moisture within the thallus and protects the algal photobiont (Beazley et al. 2002).

In *Acarosporaceae*, pruinose species commonly occur in *Acarospora* and *Sarcogyne*, and some of these had been divided into two groups, the *A. strigata* group and the *A. glaucocarpa* group by morphological characteristics, particularly by the distribution of pruina. The *A. strigata* group is characterized by heavily pruinose areoles with deep fissures cleaving the cortex, e.g., *A. strigata* and *A. tianshanica* (Nurtai et al. 2017). The *A. glaucocarpa* group is characterized by brown squamules with usually white margins and large, solitary apothecia that are typically pruinose, e.g., *A. glaucocarpa* and *S. wheeleri* (Knudsen et al. 2020). In addition, during our research on *Acarosporaceae* species with pruina, we found many more types of pruina: some species have pruina only on the thallus margin, with no pruina on the disc, e.g., *A. pulvinata* (Magnusson 1940); some lecideine species especially in *Sarcogyne* have pruina only on the apothecial disc, e.g., *S. pruinosa* and *S. belarusensis* (Knudsen et al. 2023); some species exhibit two types (pruinose or epruinose), e.g., *A. americana* and *A. cervina* (Knudsen et al. 2021).

Before this study, 18 pruinose species of *Acarosporaceae* had been reported from China: *Acarospora aeginaica*, *A. americana*, *A. cervina*, *A. glaucocarpa*, *A. glypholecioides*, *A. interrupta*, *A. mongolica*, *A. nodulosa*, *A. pulvinata*, *A. stapfiana*, *A. strigata*, *A. tianshanica*, *A. turpanensis*, *A. umbilicata*, *A. veronensis*, *Glypholecia qinghaiensis*, *G. scabra*, and *Sarcogyne pruinosa* (Magnusson 1940, 1944; Obermayer 2004; Li et al. 2007; Sahedat et al. 2015; Abdulla and Nurtai 2018; Nurtai et al. 2019, 2025; Wang et al. 2019; Wen et al. 2019; Yin et al. 2023; Abdulla 2025). Most of these were recorded from northwestern China, particularly from Xinjiang.

During the study on *Acarosporaceae* in northwestern China, we found and described three new pruinose species, *Sarcogyne bayingolinensis*, *S. parasitica*, and *S. yiliensis*, and one new record, *Acarospora iqbalii*. In addition, we found another chemical type (containing no secondary metabolites) in *A. tianshanica*, whereas this species was originally described as producing gyrophoric acid in China. *Acarospora glaucocarpa* was identified only by morphological characteristics when it was first reported from China; we recollected this species and provided new sequences. These taxonomic results are strongly supported by molecular phylogenetic analyses.

## Materials and methods

### Herbarium study

The specimens were mainly collected from Xinjiang Uyghur Autonomous Region, and deposited in the Lichen Section of the Botanical Herbarium, Shandong Normal University, Jinan, China (SDNU). The *Acarospora glaucocarpa* and some specimens of *A. tianshanica* were studied from the herbarium of the Kunming Institute of Botany (KUN). Morphological characteristics of the specimens were observed under a stereoscopic microscope (Nikon SMZ 745T). Anatomical features were examined under a compound microscope (Olympus CX21). Photos were taken using the Olympus SZX16 and BX16 microscope with DP72 camera system. The hymenial gel and subhymenium were tested for amyloid reaction with fresh undiluted IKI (Lugol’s iodine solution, 1%) (Knudsen and Kocourková 2018a). The amyloid reaction of asci was examined in IKI (Hafellner 1993). Secondary metabolites were analyzed by thin-layer chromatography (TLC) with solvent C (toluene: acetic acid = 170:30) (Orange et al. 2010).

### DNA extraction, PCR amplification and sequencing

Genomic DNA was extracted from the thalli and apothecia of dried and uncontaminated lichen specimens using the Sigma-Aldrich REDExtract-N-Amp Plant PCR Kit (St. Louis, MO, USA) following the manufacturer’s protocol. PCR amplification was performed for the internal transcribed spacer (ITS), nuclear large subunit rDNA (nuLSU), and mitochondrial small subunit rDNA (mtSSU). The respective primer sets employed were ITS1F/ITS4 (White et al. 1990; Gardes and Bruns 1993) for ITS, LR0R/LR5 (Vilgalys and Hester 1990; Rehner and Samuels 1995) for nuLSU, and mtSSU1/mtSSU3R (Zoller et al. 1999) for mtSSU. A total volume of 25 μL was used for each standard PCR reaction. The components were as follows: 12.5 μL MasterMix (Tiangen, Beijing, China), 8.5 μL ddH_2_O, 1 μL upstream primer, 1 μL downstream primer, and 2 μL DNA extract. Conditions for ITS and mtSSU: initial denaturation at 95 °C for 5 min, followed by five cycles (95 °C for 33 s, 56 °C for 30 s, and 72 °C for 30 s), then ten cycles (95 °C for 30 s, 54 °C for 30 s, and 72 °C for 30 s), and twenty cycles (95 °C for 30 s, 50 °C for 30 s, and 72 °C for 30 s) with a final extension at 72 °C for 15 min (Wang et al. 2025a). Conditions for nuLSU: initial denaturation at 98 °C for 3 min, followed by 30 cycles (98 °C for 10 s, 56 °C for 10 s, and 72 °C for 15 s), with a final extension at 72 °C for 10 min (Wang et al. 2025b). The polymerase chain reaction (PCR) products were sequenced by Sangon Biotech (Jinan, China).

### Phylogenetic analyses

A BLAST search was conducted on GenBank to identify similar sequences. All relevant sequences of *Acarosporaceae*, including the outgroup for the phylogenetic tree, were subsequently downloaded from the same database. *Pycnora sorophora* was used as the outgroup. The raw sequences were assembled and edited in Geneious Prime, and the three-locus sequences (ITS, nuLSU, and mtSSU) were aligned using the default multiple sequence alignment settings of MAFFT v. 7.490 (Katoh et al. 2005; Katoh and Standley 2013). The combined matrix included a total of 2149 characters of aligned DNA sequences from three-locus: ITS (595 bp), mtSSU (689 bp), and nuLSU (865 bp).

Phylogenetic analyses were performed using maximum likelihood (ML) and Bayesian inference (BI) (Suppl. material 1). Maximum likelihood (ML) analyses were conducted using IQ-TREE v. 1.6.12 (Nguyen et al. 2015). Bootstrap frequencies were calculated based on 1000 non-parametric bootstrap pseudo-replicates. Maximum likelihood bootstrap support values were derived from the 70% majority-rule consensus tree of all saved trees. Bayesian inference (BI) was conducted using MrBayes v. 3.2.7 with two independent runs of 2,000,000 generations each. The best fit model for each partition was determined using PartitionFinder 2 (Lanfear et al. 2016); based on the results, we applied the SYM+I+G model to all three partitions (ITS, nuLSU, and mtSSU). Trees were sampled at intervals of 1,000 generations, with the initial 25% discarded as burn-in. The remaining 75% of the sampled trees were used to construct a consensus tree. Bootstrap support (BS) values of ≥ 70% and posterior probabilities (PP) of ≥ 0.95 were interpreted as indicative of significant support. The generated phylogenetic trees were visualized using FigTree v. 1.4.4 (Rambaut 2016).

## Results and discussion

**Figure 1.**
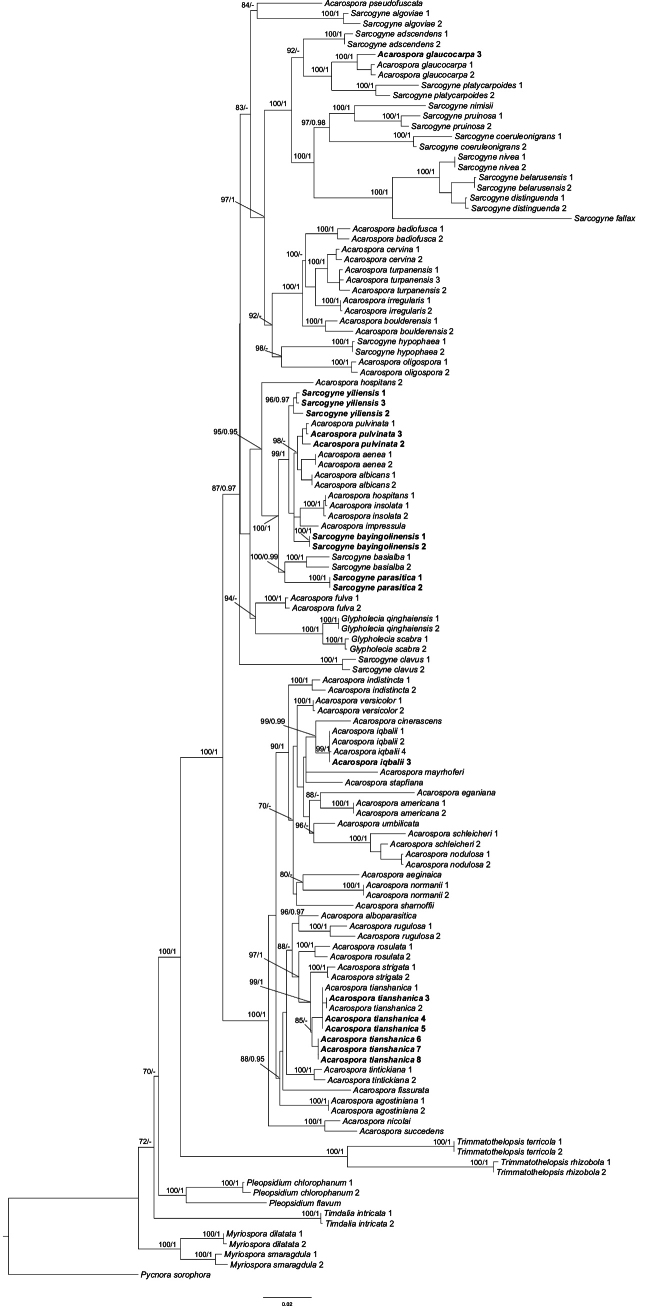
Phylogenetic tree of *Acarosporaceae* inferred from Maximum likelihood (ML) analyses based on combined ITS, mtSSU, and nuLSU sequences. *Pycnora sorophora* was used as outgroup. Maximum likelihood bootstrap support values (BS ≥ 70%) and Bayesian posterior probability (PP ≥ 0.95) are indicated above branches (BS/PP). Newly generated sequences are shown in bold.

The phylogenetic tree included a total of 327 sequences from 65 species, comprising 125 ITS, 112 mtSSU, and 90 nuLSU sequences (Suppl. material 2), of which 17 ITS, 16 mtSSU, and 10 nuLSU sequences were newly generated in this study. The ML and BI trees showed similar topologies, so only the ML tree is provided here as Fig. 1. Six genera of *Acarosporaceae* and one outgroup (*Pycnora sorophora*) were selected for the phylogenetic tree, including two non-monophyletic genera: *Acarospora* and *Sarcogyne* (with *Glypholecia* nested within), and four monophyletic genera: *Myriospora*, *Pleopsidium*, *Timdalia* and *Trimmatothelopsis*. Three new species were recovered as single lineages in *Sarcogyne* clade, and with high support values. The BS/PP values for the lineages of *S. bayingolinensis* and *S. parasitica* were 100/1, and that of *S. yiliensis* was 96/0.97.

In the phylogenetic tree, *Sarcogyne bayingolinensis* and *S. yiliensis* were recovered in the same clade with *Acarospora aenea*, *A. albicans*, *A. hospitans*, *A. impressula*, *A. insolata*, and *A. pulvinata. Sarcogyne bayingolinensi*s differs from other species in the same clade by its lobulate margin, and pruina which are mainly distributed in the center of the areolate thallus. *Sarcogyne yiliensis* differs from *A. aenea*, *A. albicans*, *A. impressula* and *A. pulvinata* by its low hymenium (50–60 μm vs. ≥ 80 μm) (Wen et al. 2019; Knudsen et al. 2021; Nurtai et al. 2025). *Acarospora hospitans* and *A. insolata* differ from *S. yiliensis* by their flat or moderate areoles (vs. irregularly bullate thallus), and thallus surface without fissures (vs. obvious fissures around apothecia) (Park et al. 2023). *Sarcogyne parasitica* was sister to *S. basialba*, but the new species differs from *S. basialba* by its areolate to subsquamulose thallus (vs. endosubstratal or endolithic thallus) (Knudsen et al. 2025a).

As discussed in the introduction, the pruinose species in *Acarosporaceae* could be divided into many groups. Although the morphological differences between these groups are distinct, molecular phylogenetic analyses have shown that these groups do not form distinct clades, indicating that the pruinose morphology has evolved multiple times independently, likely as a result of convergent adaptation.

### Taxonomy

#### New species

##### Sarcogyne bayingolinensis

Taxon classificationFungiAcarosporalesAcarosporaceae

J.X. Wang, F.H. Liang & L. Hu sp. nov.

6981F6F7-0383-5D87-99AC-6434A7A51D3A

863907

Fig. 2

###### Chinese name.

巴音郭楞网盘衣 (Ba Yin Guo Leng Wang Pan Yi).

**Figure 2.**
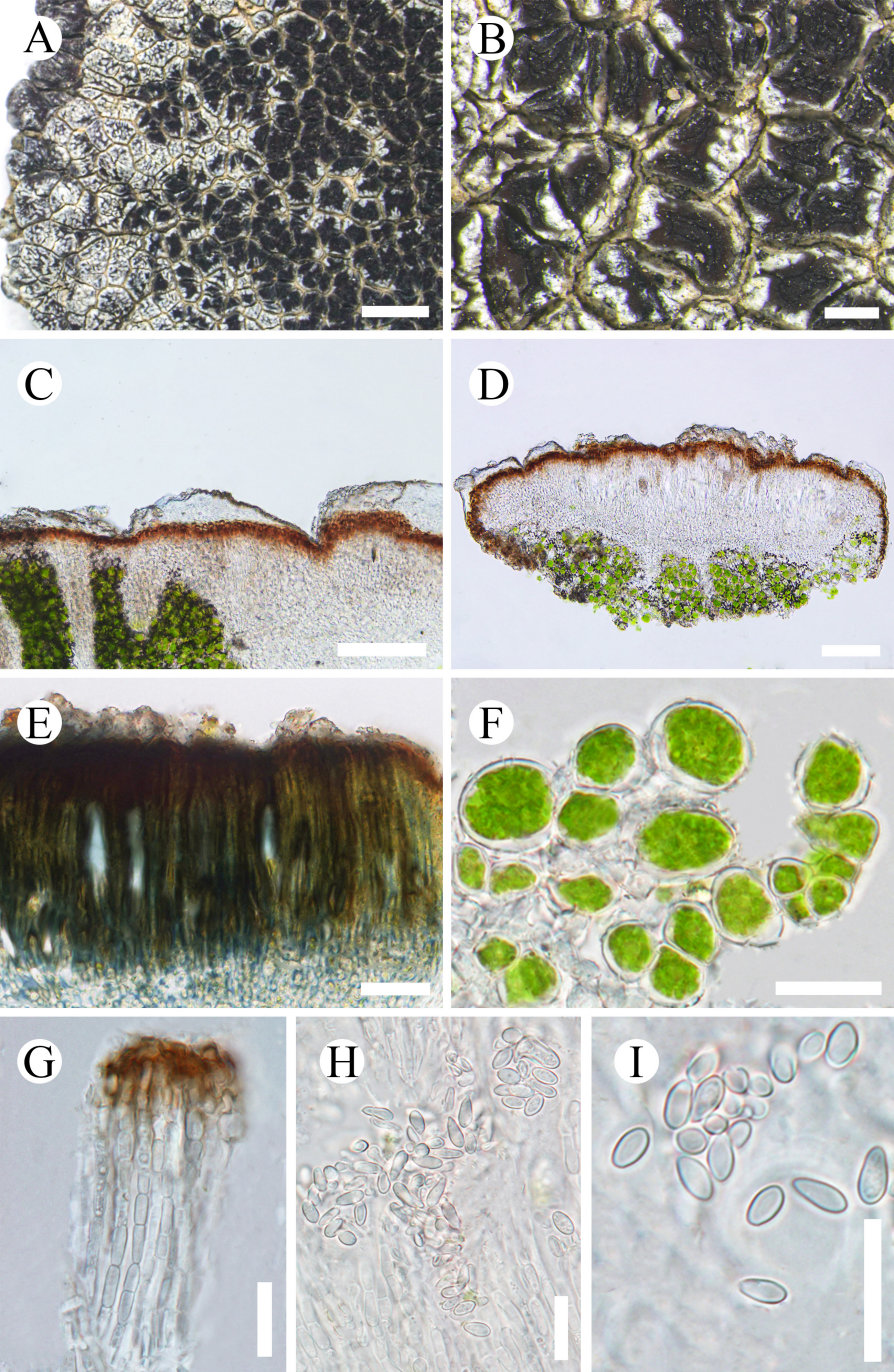
*Sarcogyne bayingolinensis* (holotype, 20240362 SDNU). **A**. Thallus with apothecia; **B**. Apothecia; **C**. Section of cortex; **D**. Section of apothecium; **E**. Hemiamyloid reaction of hymenium; **F**. Algal cells; **G**. Paraphyses; **H, I**. Ascospores. Scale bars: 1 mm (**A**); 200 μm (**B**); 50 μm (**C, D**); 20 μm (**E**); 10 μm (**F–I**).

###### Diagnosis.

Similar to *Acarospora tintickiana* but differs in having dark brown thallus (vs. usually pale brown), and in producing no secondary metabolites (vs. gyrophoric acid).

###### Type.

China • Xinjiang Uyghur Autonomous Region: Bayingolin Mongol Autonomous Prefecture, Dabancheng to Bayinbrook, beside National highway 321, 43°6'7.9"N, 84°52'34.3"E, alt. 2503 m, on calcareous rock, 16 Jul. 2024, J.X. Wang et al. 20240362 (SDNU, holotype).

###### Etymology.

The epithet bayingolinensis is derived from Bayingolin Mongol Autonomous Prefecture in Xinjiang Uyghur Autonomous Region, China, where the type specimen was collected.

###### Description.

Hypothallus endosubstratal, no algae observed. Thallus of areoles, 0.4–1.8 mm wide, 0.2–0.5 mm thick, becoming elevated by a mycelial base 1–3 mm thick, angular to irregular, contiguous, marginal areoles can be prolongated and lobate, replicating by division. Upper surface white or dark brown, slightly to densely pruinose, usually epruinose around the disc, rugulose, with some fissures. lower surface white. Epicortex ca. 10 μm. Cortex 40–100 μm thick, upper layer reddish brown, 10–20 μm thick, lower layer hyaline. Algal layer 75–125 μm thick, interrupted by hyphal bundles, algal cells 10–15 μm wide. Medulla white, 200–400 μm thick, hyphae obscure in water, intricate and gelatinized. Apothecia one to three per areole, deeply immersed, disc dark brown to black, epruinose, elongate and slit-like. Parathecium ca. 10 μm, merging with cortex. Hymenium 90–110 μm tall, epihymenium reddish brown, 25–50 μm tall, paraphyses 2–3 μm wide, apices slightly expanded in brown gel caps, hymenial gel IKI+ light blue, euamyloid. Asci narrowly clavate, 70–80 × 15–25 μm, ascospores several hundred per ascus, variable, 4–6(–8) × 2–3(–4) μm. Subhymenium 40–50 μm tall, IKI+ blue, euamyloid. Hypothecium indistinct to 10 μm thick. Pycnidia not observed. Chemistry: not producing secondary metabolites.

###### Habitat and distribution.

This new species is currently known only from its type locality. The specimens were collected from calcareous rock on a sunny slope beside a national highway, at an elevation of 2503 m.

###### Notes.

*Sarcogyne bayingolinensis* is morphologically similar to *Acarospora cinerascens*, *A. iqbalii*, *A. versicolor*, *A. tianshanica*, and *A. tintickiana. Acarospora cinerascens* differs from the new species by having the entire areole surface pruinose, uninterrupted algal layer, and longer asci (100–130 × 15–25 μm vs. 70–80 × 15–25 μm) (Knudsen et al. 2015). *Acarospora iqbalii* differs in its pruinose apothecial disc (vs. disc epruinose), lower subhymenium (20–40 μm vs. 40–50 μm), and larger asci (110–160 × 28–40 μm vs. 70–80 × 15–25 μm) (Iqbal et al. 2025). *Acarospora versicolor* differs in its thinner cortex (20–40 μm vs. 40–100 μm), uninterrupted algal layer, and round apothecial disc (Knudsen et al. 2015). *Acarospora tianshanica* differs in its uninterrupted algal layer, higher hymenium (120–150 μm vs. 90–110 μm), and longer asci (81–122 × 12–20 μm vs. 70–80 × 15–25 μm) (Nurtai et al. 2017). *Acarospora tintickiana* differs in its usually pale brown thallus (vs. dark brown) and in producing gyrophoric acid (vs. no secondary metabolites) (Leavitt et al. 2018).

###### Additional specimens examined.

China • Xinjiang Uyghur Autonomous Region: Bayingolin Mongol Autonomous Prefecture, Dabancheng to Bayinbrook, beside National highway 321, 43°6'7.9"N, 84°52'34.3"E, alt. 2503 m, on calcareous rock, 16 Jul. 2024, J.X. Wang et al. 20240379 (SDNU).

##### Sarcogyne parasitica

Taxon classificationFungiAcarosporalesAcarosporaceae

J.X. Wang, F.H. Liang & L. Hu sp. nov.

AAE24F6C-0ED1-5B5B-A692-D34DB2EEF888

863908

Fig. 3

###### Chinese name.

寄生网盘衣 (Ji Sheng Wang Pan Yi).

**Figure 3.**
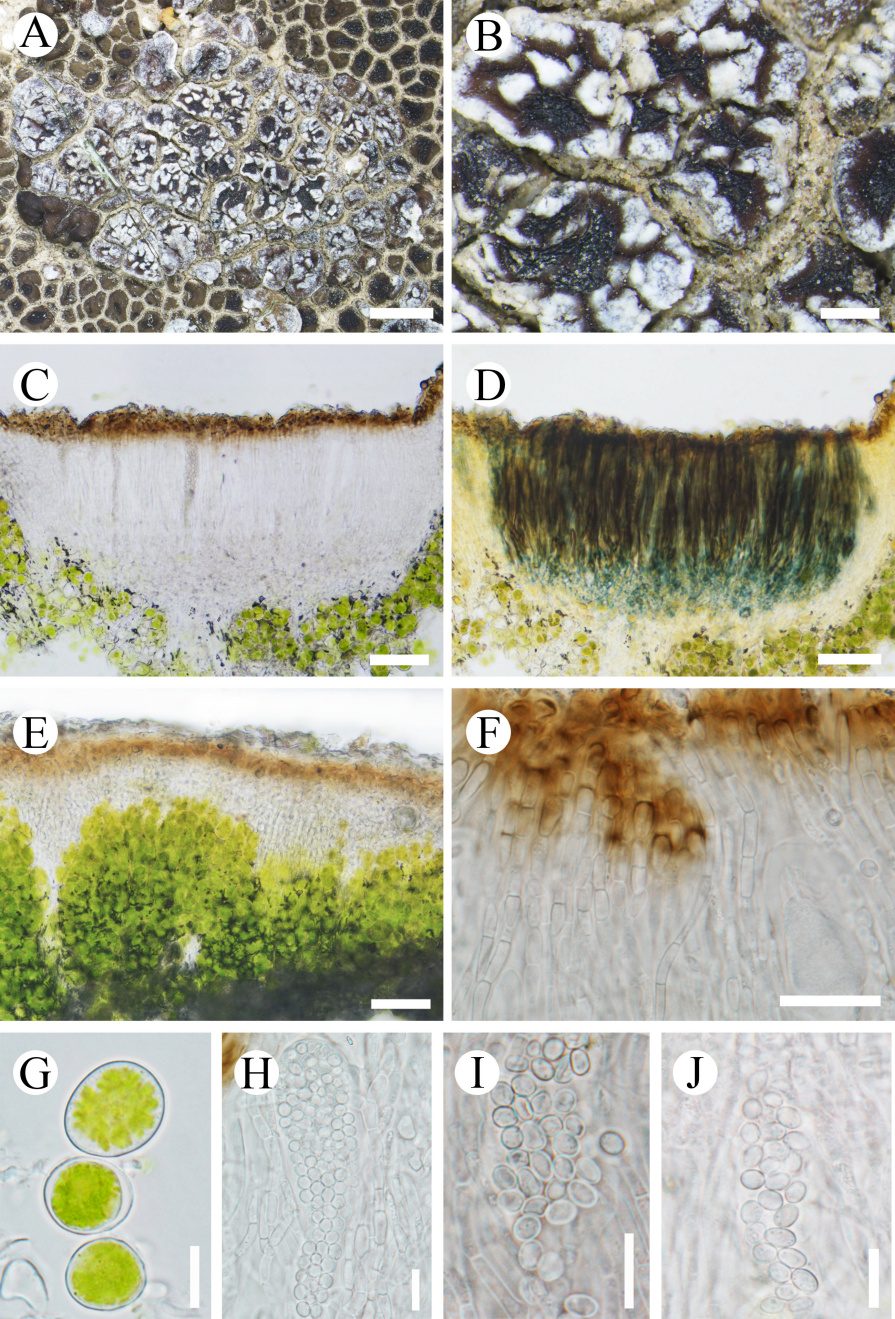
*Sarcogyne parasitica* (holotype, 20241125A SDNU). **A**. Thallus with apothecia; **B**. Apothecia; **C**. Section of apothecium; **D**. Hemiamyloid reaction of hymenium; **E**. Section of cortex; **F**. Paraphyses; **G**. Algal cells; **H**. Asci with ascospores; **I, J**. Ascospores. Scale bars: 1 mm (**A**); 200 μm (**B**); 50 μm (**C, E**); 20 μm (**D**); 10 μm (**G–I**).

###### Diagnosis.

Similar to *Acarospora aeginaica* but differs in having taller hymenium (90–150 μm vs. 50–85 μm), in having longer and narrower asci (60–90 × 10–12 μm vs. 35–60 × 12–18 μm), and in parasitizing different species (*A. bohlinii* vs. *Aspicilia* sp.).

###### Type.

China • Xinjiang Uyghur Autonomous Region: Urumqi City, Dabancheng, Donggou Village, 43°34'40.4"N, 88°36'43.2"E, alt. 1929 m, on *Acarospora bohlinii*, 22 Jul. 2024, J.X. Wang et al. 20241125A (SDNU, holotype).

###### Etymology.

Named in reference to its parasitic habit of growing on the thallus of other lichen species.

###### Description.

Thallus subsquamulose, dispersed to contiguous, irregular, 0.5–1.5 mm wide, 400–600 μm thick, lichenicolous, scattered among the thalli of *Acarospora bohlinii*, replicating by division. Upper surface white or brownish to reddish brown, slightly to densely pruinose, with some fissures. Lower surface white. Epicortex indistinct. Cortex 50–80 μm thick, upper layer reddish brown 20–30 μm thick, lower layer hyaline, of disarticulated anticlinal hyphae, cells round to irregular, 4–6 μm wide. Algal layer 80–100 μm thick, uneven, sometimes interrupted by hyphal bundles, continuous below apothecia, algal cells 10–15 μm wide. Medulla 80–200 μm thick, hyphae obscure in water. Apothecia immersed, usually 1 to 3 per squamule, sometimes with compound apothecia, disc black, 0.1–0.5 mm wide, epruinose, deeply concave, rough, with abundant shallow fissures. Parathecium 10–30 μm wide, merging with the cortex. Hymenium 90–150 μm tall, epihymenium reddish brown, ca. 10 μm tall, paraphyses 2–3 μm wide, apices unexpanded or slightly widened in terminal brown gel cap, hymenial gel IKI+ light blue, euamyloid. Asci clavate, 60–90 × 10–12 μm, ascospores several hundred per ascus, spherical to subellipsoidal, usually 3–4 × 3 μm. Subhymenium 25–40 μm tall, IKI+ blue, euamyloid. Hypothecium indistinct to 20 μm thick. Pycnidia not observed. Chemistry: not producing secondary metabolites.

###### Habitat and distribution.

This new species is parasitic on brown *Acarospora bohlinii* and is so far known only from its type locality in Donggou Village, Xinjiang Uyghur Autonomous Region, at an elevation of 1929 m, where the holotype was collected on a sunny slope near a river.

###### Notes.

*Sarcogyne parasitica* is the first reported *Acarosporaceae* species parasitizing *Acarospora bohlinii* and is characterized by pruinose thallus, IKI+ blue euamyloid hymenial gel, spherical to subellipsoidal ascospores, and the absence of secondary metabolites. This new species is similar to *A. aeginaica*, *A. mayrhoferi*, *A. invadens* and *A. superans* by being parasitic on other lichen species. *Acarospora aeginaica* differs in its lower hymenium (50–85 μm vs. 90–150 μm) and shorter and wider asci (35–60 × 12–18 μm vs. 60–90 × 10–12 μm), and in parasitizing different species (*Aspicilia* sp. vs. *A. bohlinii*) (Nurtai et al. 2017). *Acarospora mayrhoferi* differs in its epruinose thallus, wider asci (80–90 × 18–25 μm vs. 60–90 × 10–12 μm), and narrower ascospores (3–5 × 2 μm vs. 3–4 × 3–4 μm) (Knudsen and Kocourková 2018b). *Acarospora invadens* and *A. superans* were first reported from Gansu Province and similar to this new species in morphology, whereas they differed in their epruinose areoles and different lichen hosts (*Lecanora* species) (Magnusson 1940).

###### Additional specimens examined.

China • Xinjiang Uyghur Autonomous Region: Urumqi City, Dabancheng, Donggou Village, 43°34'40.4"N, 88°36'43.2"E, alt. 1929 m, on *Acarospora bohlinii*, 22 Jul. 2024, J.X. Wang et al. 20241125B (SDNU).

##### Sarcogyne yiliensis

Taxon classificationFungiAcarosporalesAcarosporaceae

J.X. Wang, F.H. Liang & L. Hu sp. nov.

6B89B88F-6FF2-5A3B-96CE-1F34D6E4EE62

863909

Fig. 4

###### Chinese name.

伊犁网盘衣 (Yi Li Wang Pan Yi).

**Figure 4.**
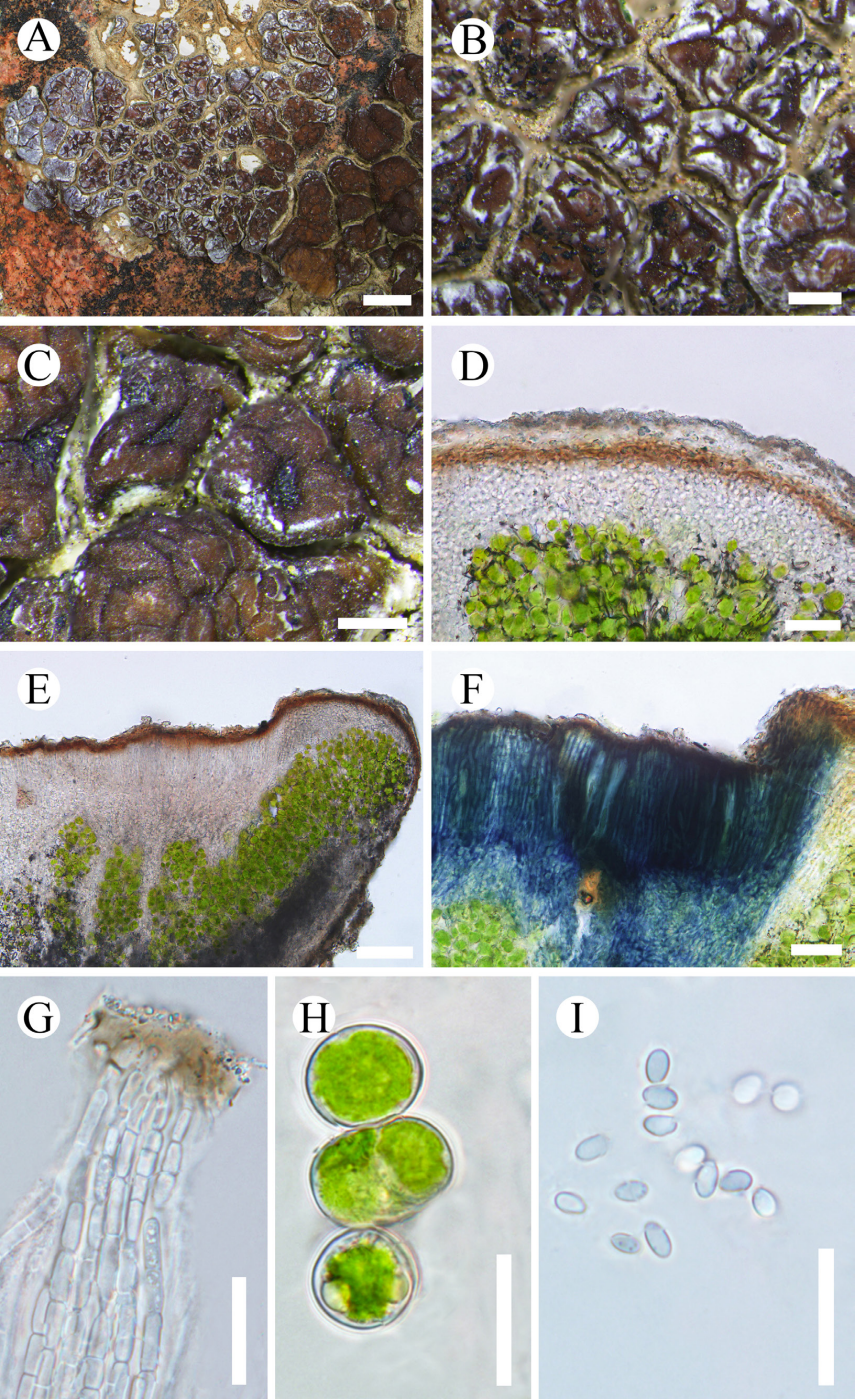
*Sarcogyne yiliensis* (holotype, 20240065 SDNU). **A**. Thallus with apothecia; **B, C**. Apothecia; **D**. Section of cortex; **E**. Section of apothecium; **F**. Hemiamyloid reaction of hymenium; **G**. Paraphyses; **H**. Algal cells; **I**. Ascospores. Scale bars: 500 μm (**A**); 200 μm (**B, C**); 50 μm (**E**); 20 μm (**D, F**); 10 μm (**G–I**).

###### Diagnosis.

Similar to *Acarospora succedens* but differs in having lower hymenium (50–60 μm vs. 80–110 μm), in having IKI+ blue euamyloid hymenial gel (vs. IKI+ blue turning red hemiamyloid), and in producing no secondary metabolites (vs. gyrophoric acid).

###### Type.

China • Xinjiang Uyghur Autonomous Region: Yili Prefecture, Xinyuan County, Xiaoerbulake Town to Nalati, beside National highway 218, 43°36'52.6"N, 82°18'0.3"E, alt. 731 m, on calcareous rock, 15 Jul. 2024, J.X. Wang et al. 20240065 (SDNU, holotype).

###### Etymology.

The specific epithet yiliensis refers to Yili Prefecture, Xinjiang Uygur Autonomous Region, the locality where the species was first collected.

###### Description.

Hypothallus endosubstratal, no algae observed. Thallus areolate to subsquamulose, dispersed to contiguous, 1–2.5 mm wide, 0.7–1.5 mm thick, irregular in shape, becoming elevated by a mycelial base, covering areas up to 3 cm or more, replicating by division. Upper surface white or brown to dark brown, shiny, uneven, pruinose or epruinose, with abundant shallow fissures. Lower surface white or brown. Epicortex indistinct to 10 μm thick. Cortex 40–60(–100) μm thick, upper layer reddish brown, 5–20 μm thick, lower layer hyaline, cells round to irregular, 3–5 μm wide. Algal layer 150–250 μm thick, uneven, interrupted by hyphal bundles, algal cells 8–12 μm wide, continuous below apothecia. Medulla white, 0.3–0.5 mm thick, hyphae obscure in water. Apothecia 1 to 4 per squamule, immersed, disc dark brown to black, 0.2–0.5 mm wide, round to irregular, epruinose. Parathecium indistinct to 10 μm wide, merging with the cortex. Hymenium 50–60 μm tall, epihymenium reddish brown, 5–15 μm tall, paraphyses 2–3 μm wide, apices unexpanded, hymenial gel IKI+ blue, euamyloid. Asci narrowly clavate, 30–40 × 10–12 μm, ascospores several hundred per ascus, ellipsoid, 4–6 × 2–3 μm. Subhymenium 50–80 μm tall, IKI+ blue. Hypothecium indistinct to 10 μm thick. Pycnidia not observed. Chemistry: not producing secondary metabolites.

###### Habitat and distribution.

This new species is currently known only from its type locality. The specimens were collected from calcareous rock on a sunny slope beside a national highway, at an elevation of 731 m.

###### Notes.

*Sarcogyne yiliensis* is similar to *Acarospora indistincta*, *A. pulvinata*, *A. sharnoffii*, and *A. succedens* in having bullate and irregular squamules. *Acarospora indistincta* differs from the new species in its lower algal layer (90–150 μm vs. 150–250 μm), higher hymenium (90–100 μm vs. 50–60 μm), larger asci (50–60 × 15–21 μm vs. 30–40 × 10–12 μm), and smaller ascospores (3–4 × 1–1.5 μm vs. 4–6 × 2–3 μm) (Knudsen et al. 2025b). *Acarospora pulvinata* differs in its pruinose thallus margin, higher hymenium (95–140 μm tall vs. 50–60 μm) and in producing gyrophoric acid (vs. not producing secondary metabolites) (Magnusson 1940; Wen et al. 2019). *Acarospora sharnoffii* differs in its lower algal layer (70–100 μm vs. 150–250 μm), higher hymenium (70–110 μm vs. 50–60 μm), and in having IKI+ blue turning red hemiamyloid hymenial gel (vs. IKI+ blue euamyloid hymenial gel) (Knudsen et al. 2025b). *Acarospora succedens* differs in its taller hymenium (80–110 μm vs. 50–60 μm), in having IKI+ blue turning red hemiamyloid hymenial gel (vs. IKI+ blue euamyloid), and in producing gyrophoric acid (vs. no secondary metabolites) (Knudsen and Kocourková 2023). *Sarcogyne yiliensis* also shares some features with *A. turpanensis*, which was first found in Xinjiang Uyghur Autonomous Region: usually pruinose thallus, interrupted algal layer and the absence of secondary metabolites, but *S. yiliensis* differs in its lower algal layer (150–250 μm vs. 260–320 μm), lower hymenium (50–60 μm vs. 75–95 μm), and smaller asci (30–40 × 10–12 μm vs. 45–55 × 10–16 μm) (Nurtai et al. 2025).

###### Additional specimens examined.

China • Xinjiang Uyghur Autonomous Region: Yili Prefecture, Xinyuan County, Xiaoerbulake Town to Nalati, beside National highway 218, 43°36'52.6"N, 82°18'0.3"E, alt. 731 m, on calcareous rock, 15 Jul. 2024, J.X. Wang et al. 20240069, 20240093 (SDNU).

#### New records

##### Acarospora iqbalii

Taxon classificationFungiAcarosporalesAcarosporaceae

M.S. Iqbal, A.U. Din, Khalid & Niazi, in Iqbal, Nadeem, Din, Khalid & Niazi, Journal of Asia-Pacific Biodiversity 18: 339 (2025)

313D8BAD-276D-555E-9467-4FF6276901D4

Fig. 5

###### Description.

Thallus crustose, areolate, sometimes becoming subsquamulose, dispersed or contiguous, even to light convex, round to irregular, 0.25–1.5 mm wide. Upper surface with thick pruina, cover the whole areoles to partly light epruinose, white or dark brown when dry, unchanged color when wet, rimose, rough and matt. Low surface white. Epicortex 10–25 μm thick. Cortex 35–50 μm thick, upper layer brown, lower layer lacking or less than 10 μm thick. Algal layer continuous, 60–160 μm thick, algal call globose, 5–8 μm diam. Medulla white, 75–300 μm. Apothecia immersed, 1–3 per areole, disc black, epruinose, initially ponctiform, then expended round to irregular, 0.1–1 mm wide, plane or concave. Parathecium 10–25 μm thick. Epihymenium pale reddish brown, 15–25 μm high. Hymenium hyaline, 50–100 μm tall, hymenium gel IKI+ blue turning red, hemiamyloid. Paraphyses 3–4 μm wide. Asci clavate, 50–55 × 10–15 μm. Ascospores broadly ellipsoid, sometimes globose, 3–4 × 2–3 μm. Subhymenium 30–50 μm thick, IKI+ blue. Hypothecium 20–25 μm thick. Pycnidia not observed. Chemistry: not producing secondary metabolites.

**Figure 5.**
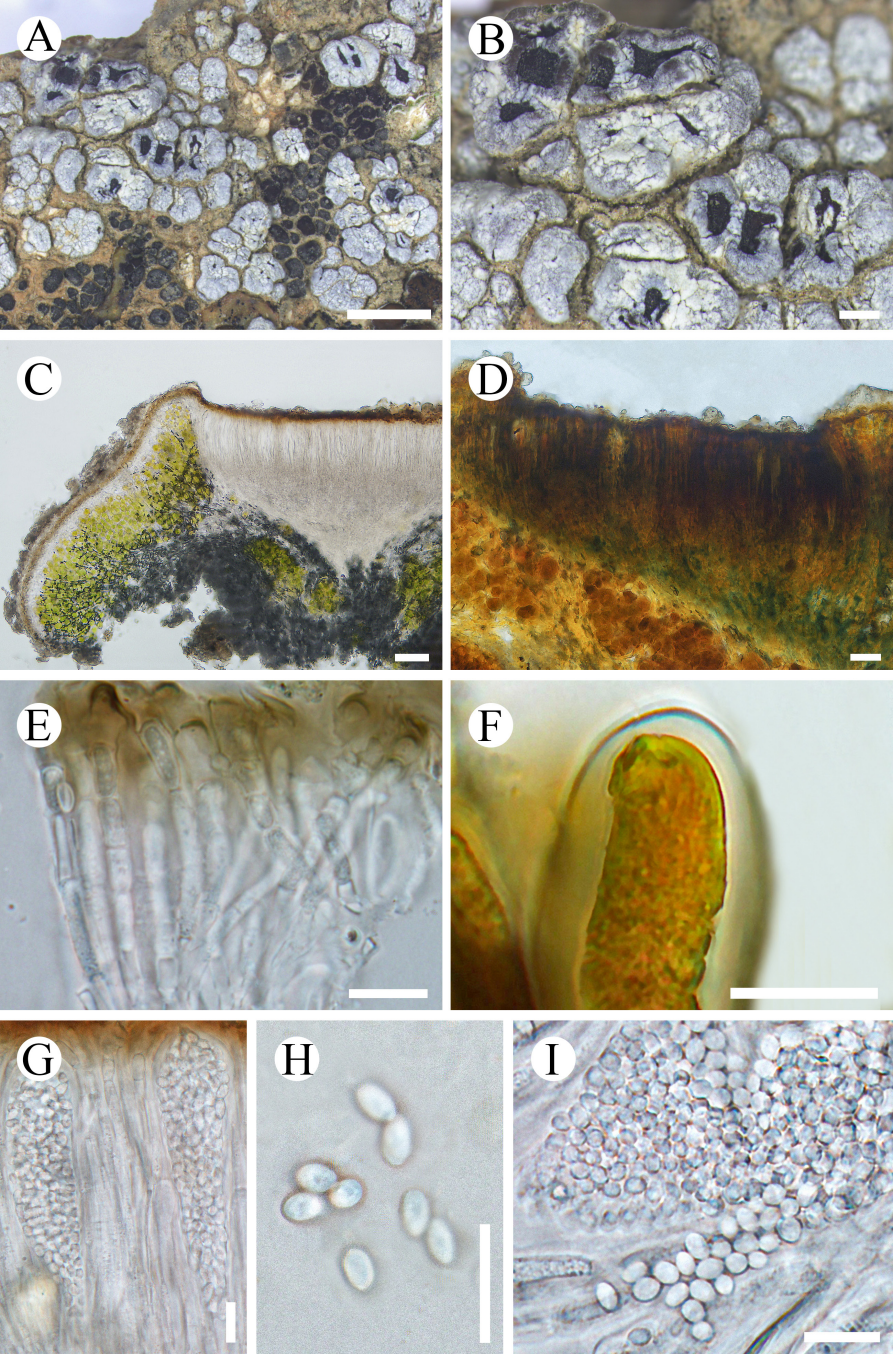
*Acarospora iqbalii* (20241418 SDNU). **A**. Thallus and substrate; **B**. Apothecia; **C**. Section of apothecium; **D**. Hemiamyloid reaction of hymenium. **E**. Paraphyses; **F**. *Acarospora*-type ascus; **G**. Multispored ascus; **H, I**. Ascospores. Scale bars: 1 mm (**A**); 200 μm (**B**); 50 μm (**C**); 20 μm (**D**); 10 μm (**E–I**).

###### Habitat and distribution.

*Acarospora iqbalii* was first known to grow on calcareous rock in cold semiarid climates, collected at 2000 m in Pakistan (Iqbal et al. 2025). In China, it was found on calcareous rock at 1958 m elevation in Xinjiang Uyghur Autonomous Region. New to China.

###### Notes.

*Acarospora iqbalii* can be easily distinguished from the other species of *Acarospora* by the entire surface of the areole covered with white pruina, fissured at the center, unchanged color when wetted with water, hymenium 50–135 μm high, and not producing secondary metabolites (Iqbal et al. 2025). The species collected from China is very similar to the Pakistan species; they both grew on calcareous rocks at similar altitudes, but the species from China had epruinose apothecia disc (vs. pruinose), and smaller asci (50–55 × 10–15 μm vs. 110–160 × 28–40 μm). *A. iqbalii* is morphologically similar to *A. tianshanica*, but the latter differs in having larger ascospores (5.5–9 × 2.5–4 μm vs. 3–4 × 2–3 μm), and in producing gyrophoric acid (vs. producing no secondary metabolites) (Nurtai et al. 2017).

###### Specimens examined.

China • Xinjiang Uyghur Autonomous Region: Urumqi, Dabancheng district, Donggou village, beside the river, 43°34'20.6"N, 88°36'11.5"E, alt. 1958 m, on calcareous rock, 22 Jul. 2024, L. Hu et al. 20241418 (SDNU).

#### Reported species

##### Acarospora glaucocarpa

Taxon classificationFungiAcarosporalesAcarosporaceae

(Ach.) Arnold, Flora 41: 311. 1858

B847F787-0B85-5C8D-B34D-79531FE32113

Fig. 6

###### Description.

Thallus areolate to squamulose, broadly attached, round to irregular, rough, with light cracks, areoles 0.5–2 mm wide, 250–350 μm thick, squamules up to 4.5 mm wide. Upper surface grayish white to light brown, slightly to densely pruinose or epruinose, sometimes only the margins of squamules with white pruina. Lower surface white. Epicortex indistinct. Cortex up to 100 μm thick, upper layer reddish brown, 10–30 μm thick, lower layer hyaline, 30–70 μm thick. Algal layer 125–200 μm thick, uneven, sometimes interrupted by hyphal bundles, algal cells 8–10 μm wide. Medulla 150–400 μm thick, often gelatinized and obscure, with or without crystals. Apothecia immersed in thallus center when young, usually one per areole, 0.2–1.5 mm wide, mature apothecia usually directly growing on substrate, or elevated above the thallus, disc reddish brown to dark brown, round, up to 4 mm wide, epruinose or pruinose, rough, concave, often lower than the margin. Parathecium 30–100 μm wide, expanding around the disc. Hymenium 60–100 μm tall, epihymenium reddish brown, 10 μm tall, paraphyses 2–3 μm wide, apices unexpanded or slightly widened to 3.5 μm in terminal brown gel cap, hymenial gel IKI+ dark blue, euamyloid. Asci clavate, 60–70 × 10–15 μm, usually poorly developed, ascospores narrow ellipsoid to ellipsoid, 3–5 × 1–2 μm. Subhymenium 20–40 μm tall, IKI+ blue, euamyloid. Hypothecium 25–50 μm thick. Pycnidia not observed. Chemistry: not producing secondary metabolites.

**Figure 6.**
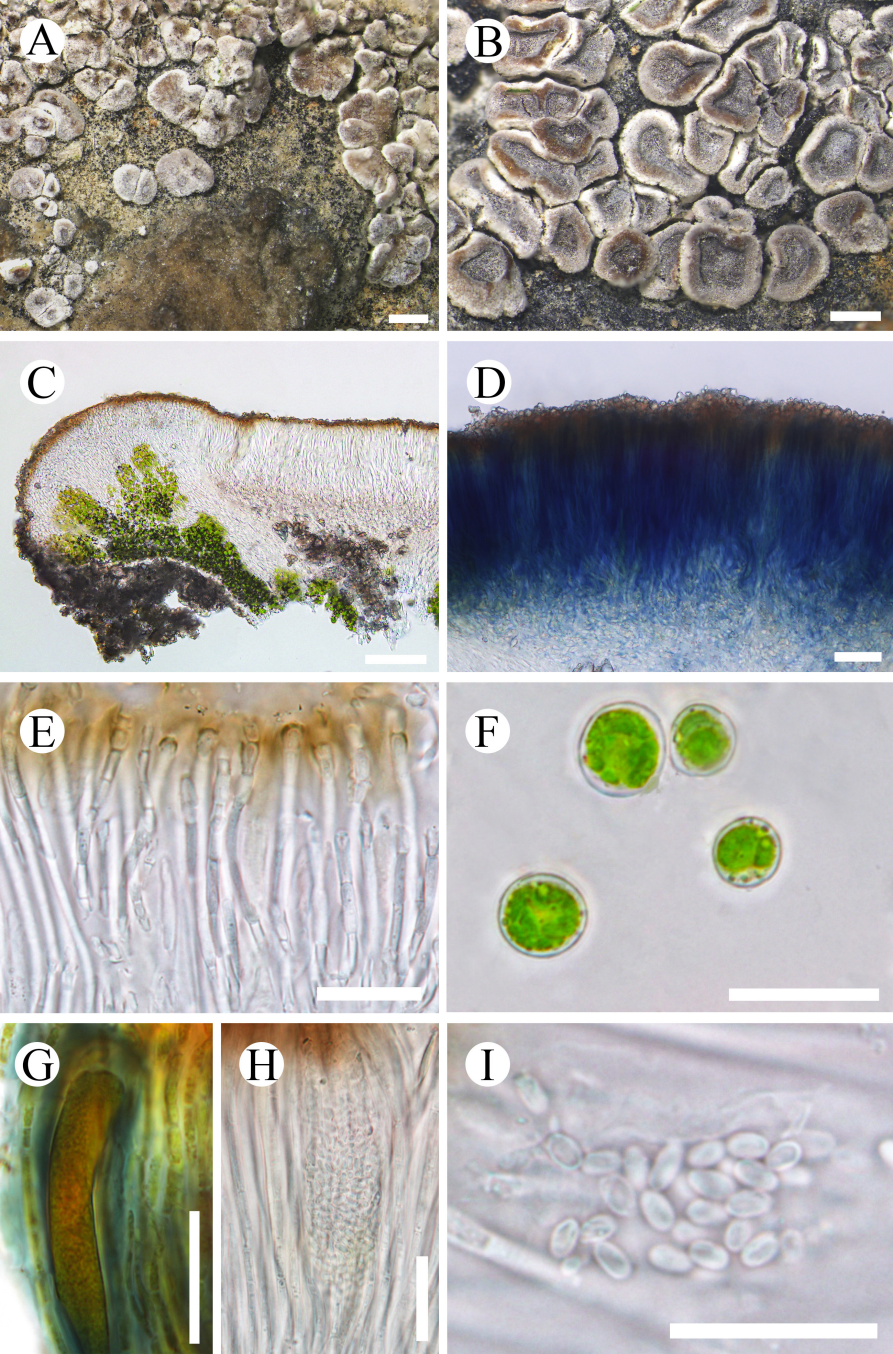
*Acarospora glaucocarpa* (24-76487 KUN). **A**. Thallus; **B**. Apothecia; **C**. Section of apothecium; **D**. Hemiamyloid reaction of hymenium; **E**. Paraphyses; **F**. Algal cells; **G**. Ascus in IKI; **H**. Asci with ascospores; **I**. Ascospores. Scale bars: 1 mm (**A**); 500 μm (**B**); 50 μm (**C**); 20 μm (**D**); 10 μm (**E–I**).

###### Habitat and distribution.

This species in China was found in Xinjiang Uyghur Autonomous Region, at an elevation of 1929 m, and in Yunnan Province, at an elevation of 4211 m.

###### Notes.

This species was first reported from Xinjiang Uyghur Autonomous Region in China only based on morphology (Li et al. 2007). We later rediscovered it in Yunnan Province; this specimen closely resembles the specimen reported from Xinjiang based on the previous description. Based on morphological, chemical and molecular data, we identified it as *Acarospora glaucocarpa*, and supplemented detailed descriptions, high-resolution appearance images, and detailed anatomical illustrations.

###### Specimens examined.

China • Yunnan Province: Deqin County, Shengping Town, 28°22'24.77"N, 99°01'15.40"E, alt. 4211 m, on calcareous rock, 12 Jul. 2024, L.S. Wang et al. 24-76487 (KUN).

##### Acarospora tianshanica

Taxon classificationFungiAcarosporalesAcarosporaceae

A. Abbas, L. Nurtai & K. Knudsen, Bryologist 120(4): 385 (2017)

E048E61F-1408-5667-B691-36D3FB1B36A8

Fig. 7

###### Description.

Thallus of areoles to subsquamules dispersed to contiguous, irregular, 0.3–1.5(–2) mm wide. Upper surface white to grayish white. Slightly to densely pruinose, with some fissures, rough. Lower surface white to brown. Epicortex 10–25 μm thick. Cortex 50–75 μm thick, upper layer brownish 5–10 μm thick, lower layer hyaline, cells round to irregular, 4–6 μm wide. Algal layer up to 150 μm thick, usually interrupted by hyphal bundles, algal cells 8–12 μm wide. Medulla up to 250 μm thick, hyphae obscure in water. Apothecia immersed, usually 1 to 3 per squamule, disc black, up to 3 mm wide, usually epruinose, rough. Parathecium 5–20(–50) μm, sometimes merging with the cortex. Hymenium 100–150 μm tall, epihymenium brown, 5–10 μm tall, paraphyses 1–3 μm wide, apices unexpanded or slightly widened in terminal brown gel cap, hymenial gel IKI+ blue to red, hemiamyloid. Asci clavate, 75–125 × 15–25 μm, ascospores narrow ellipsoid, 5–8 × 2–4 μm. Subhymenium 20–40 μm tall, IKI+ blue, euamyloid. Hypothecium 15–30 μm thick. Pycnidia not observed. Chemistry: producing gyrophoric acid or no secondary metabolites.

**Figure 7.**
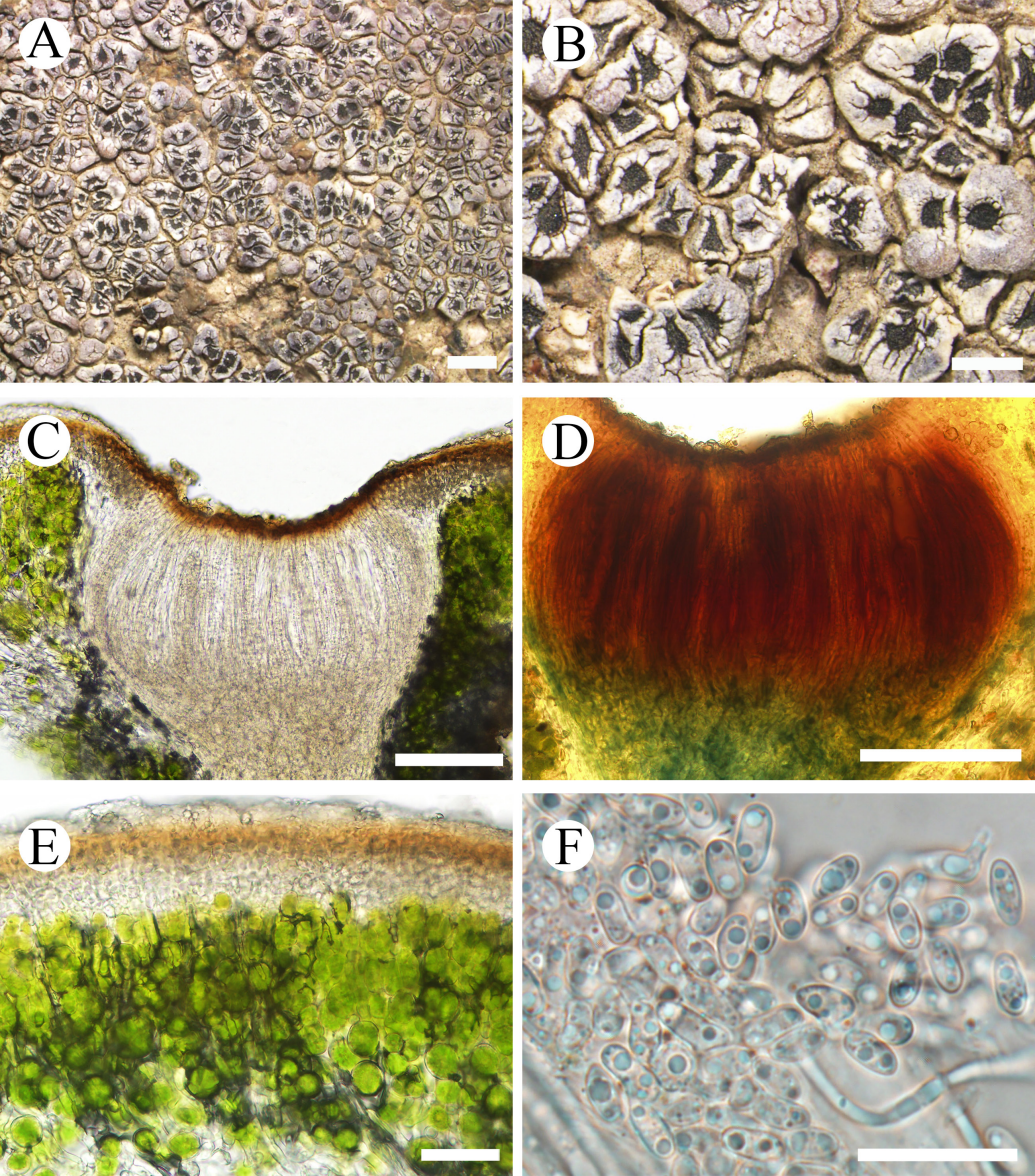
*Acarospora tianshanica* (20241407 SDNU). **A**. Thallus with apothecia; **B**. Apothecia; **C**. Section of apothecium; **D**. Hemiamyloid reaction of hymenium; **E**. Section of cortex; **F**. Ascospores. Scale bars: 1 mm (**A**); 500 μm (**B**); 50 μm (**C, D**); 20 μm (**E**); 10 μm (**F**).

###### Habitat and distribution.

*Acarospora tianshanica* was found in Gansu Province, Inner Mongolia Autonomous Region, Qinghai Province, and Xinjiang Uyghur Autonomous Region, at elevations of 1500–3000 m, on calcareous and siliceous rock in full sun. We predict this species is widely distributed in Central Asia.

###### Notes.

The holotype of *Acarospora tianshanica* was collected from Tianshan Grand Canyon, Xinjiang Uyghur Autonomous Region, China (Nurtai et al. 2017). In the protologue, this species was reported to produce gyrophoric acid. However, based on TLC analysis of numerous specimens collected from Xinjiang Uyghur Autonomous Region in this study, the number of specimens producing gyrophoric acid was approximately equal to those producing no secondary metabolites. Therefore, we have supplemented the chemical description of this species. In addition, this species was reported only growing on granite (siliceous rock), however, we collected some specimens that also grew on calcareous rock.

###### Specimens examined.

China • Gansu Province: Subei Mongol Autonomous County, Danghe Canyon, 39°27'34.81"N, 94°57'17.59"E, alt. 2376 m, on calcareous rock, 23 May 2018, L.S. Wang et al. 18-58447 (KUN); Linze County, Danxia Landform Park, 38°58'31.84"N, 100°01'06.64"E, alt. 1738 m, on calcareous rock, 29 May 2018, L.S. Wang et al. 18-58734 (KUN); • Inner Mongolia Autonomous Region: Alxa Left Banner, Helan Mountain National Nature Reserve, 38°54'56"N, 105°51'54"E, alt. 1500 m, on calcareous rock, 19 Aug. 2011, P.M. Wang 20123235 (SDNU); • Qinghai Province: Delingha City, near Chayegou Protection Station, 37°24'01.64"N, 96°35'05.74"E, alt. 2944 m, on calcareous rock, 21 May 2018, L.S. Wang et al. 18-58357 (KUN); • Xinjiang Uyghur Autonomous Region: Urumqi City, near the checkpoint of Tianshan No. 1 Glacier, 43°24'57.6"N, 87°13'11.3"E, alt. 1726 m, on calcareous rock, 20 Jul. 2024, L. Hu, Y.H. Wang & J.X. Wang 20240776, 20240800 (SDNU); Urumqi City, West Baiyanggou, 43°29'37.07"N, 87°11'41.3"E, alt. 1877 m, on calcareous rock, 20 Jul. 2024, L. Hu, Y.H. Wang & J.X. Wang 20240806, 20240816b (SDNU); near the Tianshan Glacier Station of CAS, 43°12'46.4"N, 87°7'25.3"E, alt. 2090 m, on calcareous rock, 20 Jul. 2024, L. Hu, Y.H. Wang & J.X. Wang 20240843 (SDNU); Urumqi City, Dabancheng District, Donggou Village, 43°34'40.4"N, 88°36'43.2"E, alt. 1929 m, on calcareous rock, 22 Jul. 2024, L. Hu, Y.H. Wang & J.X. Wang 20241150 (SDNU); Urumqi City, Dabancheng District, on the hillside by the river ditch of Donggou Village, 43°35'48.5"N, 88°37'49.0"E, alt. 1971 m, on calcareous rock, 22 Jul. 2024, L. Hu, Y.H. Wang & J.X. Wang 20241227 (SDNU); Urumqi City, Dabancheng District, by the river course of Donggou Village, 43°34'20.6"N, 88°36'11.5"E, alt. 1958 m, on calcareous rock, 22 Jul. 2024, L. Hu, Y.H. Wang & J.X. Wang 20241243, 20241313, 20241393, 20241397A, 20241407 (SDNU).

### Key to *Acarosporaceae* with prunia in China

**Table:** 

1	Apothecia lecanorine	**2**
–	Apothecia lecideine	***Sarcogyne pruinosa* (Obermayer 2004)**
2	On soil crust or other lichen species	**3**
–	On rock	**6**
3	On soil crust	***Acarospora nodulosa* (Wen et al. 2019)**
–	On lichen species	**4**
4	Thallus yellow beneath pruina, on species of *Caloplaca*	***A. stapfiana* (Nurtai et al. 2019)**
–	Thallus brown beneath pruina, on other hosts	**5**
5	On *A. bohlinii*, hymenium 90–150 μm tall	***S. parasitica* (this paper)**
–	On species of *Aspicilia*, hymenium 50–85 μm tall	***A. aeginaica* (Nurtai et al. 2017)**
6	With gyrophoric acid	**7**
–	Without gyrophoric acid	**13**
7	Ascospores globose to subglobose, 3–5 × 3–4 μm	**8**
–	Ascospores ellipsoid, 3–9 × 1.7–4 μm	**9**
8	Thallus not peltate	***A. mongolica* (Magnusson 1944)**
–	Thallus peltate	***Glypholecia scabra* (Yin et al. 2023)**
9	Thallus low surface with rhizines	***G. qinghaiensis* (Yin et al. 2023)**
–	Thallus low surface without rhizines	**10**
10	Only thallus margin pruinose	***A. pulvinata* (Magnusson 1940; Wen et al. 2019)**
–	Thallus surface pruinose	**11**
11	Thallus squamulose, usually imbricate	***A. glypholecioides* (Magnusson 1940)**
–	Thallus areolate to subsquamulose, rarely imbricate	**12**
12	With radial fissures around apothecia, ascospores 4–5 × 1.5 μm	***A. umbilicata* (Wang et al. 2019; Knudsen et al. 2022)**
–	Without radial fissures around apothecia, ascospores 5.5–9 × 2.5–4 μm	***A. tianshanica* (Nurtai et al. 2017)**
13	Thallus surface usually without fissures	**14**
–	Thallus surface usually with fissures	**17**
14	Apothecial disc usually pruinose	***A. glaucocarpa* (Li et al. 2007; Knudsen et al. 2020)**
–	Apothecial disc usually epruinose	**15**
15	Algal layer often interrupted by hyphal bundles	***A. cervina* (Knudsen et al. 2021; Abdulla 2025)**
–	Algal layer continuous, not interrupted by hyphal bundles	**16**
16	Thallus usually lobulate around margin, and with parathecial crowns around disc	***A. americana* (Knudsen et al. 2011; Sahedat et al. 2015)**
–	Thallus usually not lobulate around margin, and without parathecial crowns around disc	***A. veronensis* (Magnusson 1929; Knudsen et al. 2021)**
17	Thallus determinate, center areolate, margin lobulate	***S. bayingolinensis* (this paper)**
–	Thallus indeterminate, round to angular	**18**
18	Hymenial gel IKI+ blue turning red	**19**
–	Hymenial gel IKI+ blue	**21**
19	Thallus usually lobulate around margin	***A. tianshanica* (Nurtai et al. 2017)**
–	Thallus usually not lobulate around margin	**20**
20	Thallus surface convex, obvious fissures around apothecia	***A. strigata* (Knudsen et al. 2008; Nurtai et al. 2017)**
–	Thallus surface even to concave, slight fissures around apothecia	***A. iqbalii* (Iqbal et al. 2025)**
21	Hymenium ≤ 60 μm	***S. yiliensis* (this paper)**
–	Hymenium > 70 μm	**22**
22	Algal layer uninterrupted by hyphal bundles	***A. interrupta* (Nurtai et al. 2017)**
–	Algal layer interrupted by hyphal bundles	***A. turpanensis* (Nurtai et al. 2025)**

## Supplementary Material

XML Treatment for Sarcogyne bayingolinensis

XML Treatment for Sarcogyne parasitica

XML Treatment for Sarcogyne yiliensis

XML Treatment for Acarospora iqbalii

XML Treatment for Acarospora glaucocarpa

XML Treatment for Acarospora tianshanica

## Additional information

### Artificial Intelligence (AI) use

The authors accept full responsibility for the content of the manuscript, including the disclosure of any use of AI.

No AI tools were used in the preparation of this manuscript.

### Author ORCIDs

F. Liang https://orcid.org/0009-0000-7210-0607

J. Wang https://orcid.org/0009-0008-6376-4071

L. Hu https://orcid.org/0000-0002-9451-383X

C. Miao https://orcid.org/0009-0003-6450-8145

H. Si https://orcid.org/0000-0003-0585-3478

R. Chang https://orcid.org/0000-0001-6613-9062

L. Zhang https://orcid.org/0000-0001-8011-4451

X. Wang https://orcid.org/0000-0003-2166-6111

L. Wang https://orcid.org/0000-0003-3721-5956

Z. Zhao https://orcid.org/0009-0007-2970-9231

### Data availability

All of the data that support the findings of this study are available in the main text or Supplementary Information.

## Supplementary materials

Supplementary material 1 Phylogenetic tree constructed through BI analyses based on ITS, nuLSU, and mtSSU for *Acarosporaceae* Data type pdf Explanation note *Pycnora sorophora* was used as the outgroup. Only significant values (higher than 0.95 PP) are shown. https://binary.pensoft.net/file/1721656This dataset is made available under the Open Database License (http://opendatacommons.org/licenses/odbl/1.0/). The Open Database License (ODbL) is a license agreement intended to allow users to freely share, modify, and use this Dataset while maintaining this same freedom for others, provided that the original source and author(s) are credited.Author: Fuhui Liang

Supplementary material 2 A list of sampled specimens included in the molecular phylogeny Data type docx https://binary.pensoft.net/file/1721657This dataset is made available under the Open Database License (http://opendatacommons.org/licenses/odbl/1.0/). The Open Database License (ODbL) is a license agreement intended to allow users to freely share, modify, and use this Dataset while maintaining this same freedom for others, provided that the original source and author(s) are credited.Author: Fuhui Liang
