# Supplementary figures and images for: Discovering the diversity of Acarosporaceae with pruina in China
Source: MycoKeys. 2026 Jul 21;137:175–97. doi: 10.3897/mycokeys.137.201158 (PMC13416818; doi:10.3897/mycokeys.137.201158)

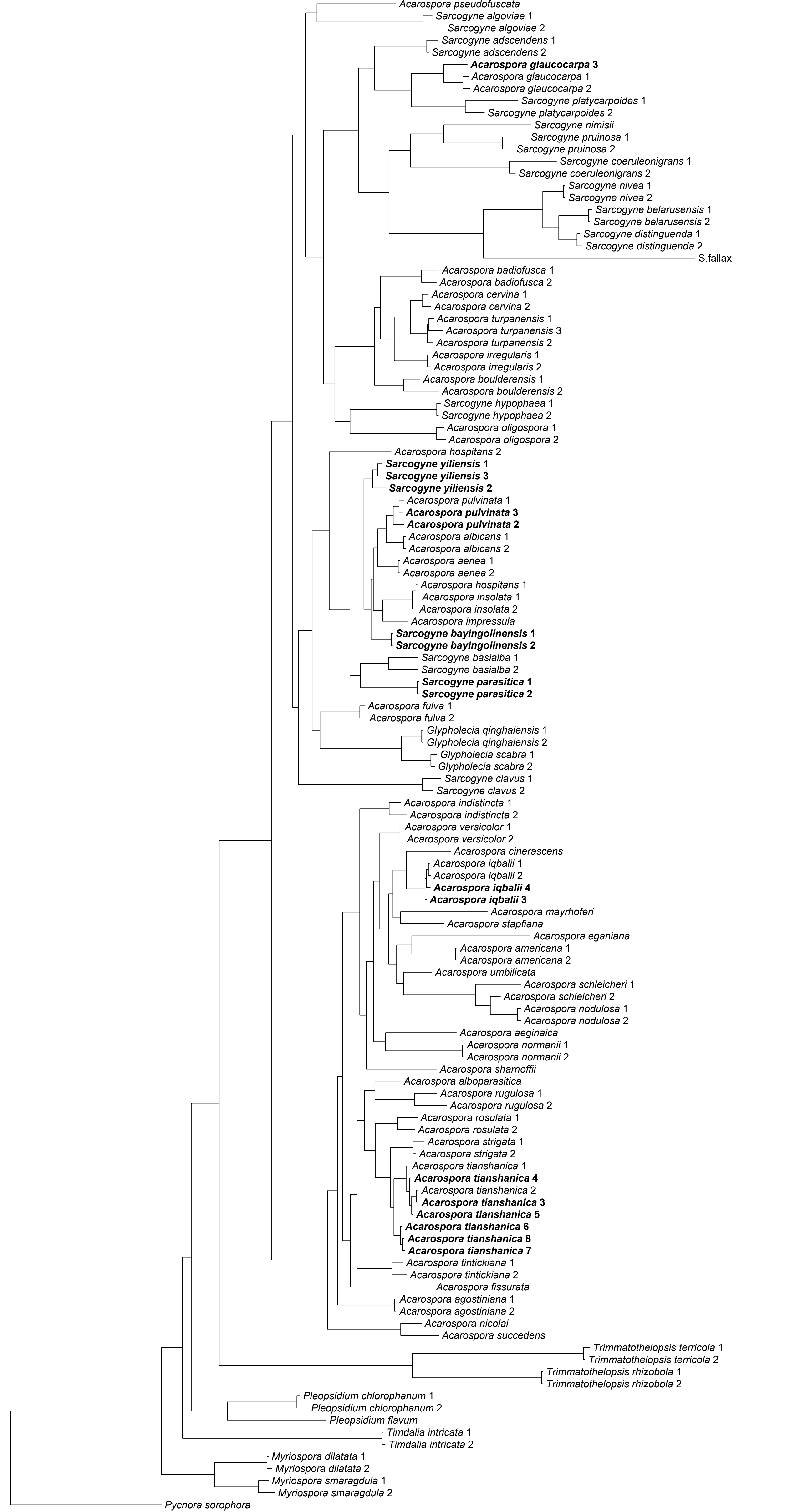

Supplement: Supplementary material 1 — Phylogenetic tree constructed through BI analyses based on ITS, nuLSU, and mtSSU for Acarosporaceae [file mycokeys-137-175-s001.pdf]
