# Supplementary material for: Discovering the diversity of Acarosporaceae with pruina in China
Source: MycoKeys. 2026 Jul 21;137:175–97. doi: 10.3897/mycokeys.137.201158 (PMC13416818; doi:10.3897/mycokeys.137.201158)
Supplement: Supplementary material 2 — A list of sampled specimens included in the molecular phylogeny [file mycokeys-137-175-s002.docx]

**Supplemental Materials 2.** Table of sampled specimens included in the molecular phylogeny.

| **Species** | **Country** | **Voucher** | **ITS** | **mtSSU** | **nuLSU** |
| --- | --- | --- | --- | --- | --- |
| *Acarospora aeginaica* | Greece | Sipman 59972 | MZ262721 | MZ262733 | MZ262743 |
| *A. aenea* 1 | China | Abbas et al. 20119810 (XJU) | MK614817 | MK637551 | MK636531 |
| *A. aenea*2 | China | Abbas et al. 20070410 (holotype | MK614818 | MK637535 | - |
| *A. agostiniana* 1 | USA | Knudsen 19272 & Kocourková (PRM) | OK142738 | OK032123 | - |
| *A. agostiniana* 2 | New Mexico | Knudsen 19236 (PRM) | ON707085 | ON715682 | ON725172 |
| *A. albicans* 1 | Pakistan | LAH38273 | PQ165150 | - | - |
| *A. albicans* 2 | Pakistan | LAH38274 | PQ165151 | - | - |
| *A. alboparasitica* | USA | Leavitt S. 18–018 (BYU-C) | PV873177 | PV883311 | PV883290 |
| *A. americana* 1 | California | Dart 1526.1 (hb. K&k) | OP162349 | OP177737 | OP216668 |
| *A. americana* 2 | California | Dart 750 (hb. K&K) | OP162350 | OP177738 | OP216669 |
| *A. badiofusca* 1 | China | Abbas et al. 201183111 (XJU) | MK614809 | MK637548 | MK636527 |
| *A. badiofusca* 2 | Sweden | Nordin & Owe-Larsson 36 (UPS) | LN810763 | LN810888 | LN810763 |
| *A. boulderensis* 1 | Kansas | Morse 15147 (SBBG) | OP162359 | OP177746 | OP216677 |
| *A. boulderensis* 2 | Minnesota | Wetmore 37667 (GZU) | OP162391 | OP177775 | OP216708 |
| *A. cervina* 1 | Czech Republic | Kocourková (PRM 952325) | OK142756 | OK032141 | - |
| *A. cervina* 2 | Sweden | Westberg SAR200 (S) | LN810765 | LN810890 | LN810765 |
| *A. cinerascens* | China | 20150353 | MK614821 | - | - |
| *A. eganiana* | USA | Haimo Pölzl s.n. (GZU) | OM522314 | OM522316 | OM572527 |
| *A. fissurata* | USA | Kocourková 10250 (PRM) | OK142742 | OK032127 | - |
| *A. fulva* 1 | China | Abbas et al. 20141065-b (XJU) | MK500841 | MK508865 | MK530411 |
| *A. fulva* 2 | China | Abbas et al. 2014052A (XJU) | MK500857 | MK509011 | MK530416 |
| *A. glaucocarpa* 1 | Sweden | Westberg SAR08 (LD) | LN810768 | LN810893 | LN810768 |
| *A. glaucocarpa* 2 | Sweden | Westberg WE23 (LD) | LN810769 | LN810894 | LN810769 |
| ***A. glaucocarpa* 3** | **China** | **KUN 24-76487** | **PZ403674** | **PZ403691** | - |
| *A. hospitans* 1 | Norway | Westberg 08-234 (S) | LN810775 | LN810900 | LN810775 |
| *A. hospitans* 2 | South Korea | KHL0002773 (KH) | OQ629810 | OQ641683 | - |
| *A. impressula* | Norway | Westberg 08-107 (S F121708) | LN810776 | LN810901 | LN810776 |
| *A. indistincta* 1 | California | Knudsen 5782 (SBBG) | ON707096 | ON715691 | ON725180 |
| *A. indistincta* 2 | California | Knudsen 12772 (SBBG) | ON707099 | ON715695 | ON725184 |
| *A. insolata* 1 | Sweden | Westberg 06-022 (LD) | LN810777 | LN810902 | LN810777 |
| *A. insolata* 2 | Czech Republic | Wagner s.n. (hb. Wagner) | OK142743 | OK032128 | - |
| *A. iqbalii* 1 | Pakistan | LAH38278 | PQ037951 | - | - |
| *A. iqbalii* 2 | Pakistan | LAH38272 | PQ037950 | - | - |
| *A.* iqbalii 3 | China | SDNU 20241418 | PV989560 | PV989570 | PV989565 |
| ***A.*** iqbalii 4 | **China** | **KUN 18-59598** | **PZ403675** | **PZ403692** | **-** |
| *A. irregularis* 1 | China | Abbas et al. 2014003A (XJU) | MK614814 | MK637550 | MK636529 |
| *A. irregularis* 2 | China | Abbas et al. 20081040 (XJU) | MK614815 | MK637549 | MK636528 |
| *A. mayrhoferi* | Italy | Kocourková 9007 | MZ262719 | MZ262731 | MZ262742 |
| *A. nicolai* | Kansas | Morse 16138 (S) | LN810785 | LN810910 | LN810785 |
| *A. nodulosa* 1 | Spain | Westberg 10-215 (S) | LN810789 | LN810914 | LN810789 |
| *A. nodulosa* 2 | Spain | Westberg SCIN032 (S) | LN810788 | LN810913 | LN810788 |
| *A. normanii* 1 | Norway | Westberg 12-011 | LN810786 | LN810911 | LN810786 |
| *A. normanii* 2 | Sweden | Westberg 3110 (LD) | LN810787 | LN810912 | LN810787 |
| *A. oligospora* 1 | Norway | Westberg 08-106 (S F121705) | LN810791 | LN810916 | LN810791 |
| *A. oligospora* 2 | Sweden | Westberg 09-659 & Tibell (S) | LN810792 | LN810917 | LN810792 |
| *A. pseudofuscata* | Greece | Sipman 11/2013-13 (B) | MZ262718 | MZ262730 | MZ262741 |
| *A. pulvinata* 1 | China | Abbas 20157528-b | MK129269 | - | - |
| ***A. pulvinata* 2** | **China** | **SDNU 20240530** | **PZ403676** | **PZ403693** | **PZ403707** |
| ***A. pulvinata* 3** | **China** | **SDNU 20241401** | **PZ403677** | **PZ403694** | **-** |
| *A. rosulata* 1 | USA | Knudsen 9509 (S F256011) | LN810796 | LN810921 | LN810796 |
| *A. rosulata* 2 | Norway | Westberg 08-193 (S) | LN810797 | LN810922 | LN810797 |
| *A. rugulosa* 1 | Norway | Westberg 08-119 (S) | LN810798 | LN810923 | LN810798 |
| *A. rugulosa* 2 | Norway | Westberg 10-099 (S) | LN810799 | LN810924 | LN810799 |
| *A. schleicheri* 1 | China | Obermayer 2919 (UPS L-070426) | LN810800 | LN810925 | LN810800 |
| *A. schleicheri* 2 | California | Knudsen 15559 (SBBG) | ON794213 | ON787697 | - |
| *A. sharnoffii* | California | Sharnoff 4107 (SBBG) | ON707101 | ON715697 | ON725185 |
| *A. stapfiana* 1 |  |  | MF134870 | - | - |
| *A. strigata* 1 | USA | Knudsen 9505 (S F256017) | LN810805 | LN810930 | LN810805 |
| *A. strigata* 2 | USA | Knudsen 9408 (S F223070) | MH555408 | MH555425 | MH555363 |
| *A. succedens* | USA | Knudsen 12876 (SBBG) | OR887204 | - | - |
| *A. tianshanica 1* | China | 20179371 | MK503500 | - | - |
| *A. tianshanica* 2 | China | 20130069 | MG838742 | - | - |
| ***A. tianshanica* 3** | **China** | **SDNU 20241243** | **PZ403679** | **PZ403696** | **PZ403708** |
| ***A. tianshanica* 4** | **China** | **SDNU 20241150** | **PZ403678** | **PZ403695** | - |
| ***A. tianshanica* 5** | **China** | **SDNU 20241313** | **PZ403680** | **PZ403697** | **PZ403709** |
| ***A. tianshanica* 6** | **China** | **SDNU 20241397A** | **PZ403681** | **PZ403698** | - |
| ***A. tianshanica* 7** | **China** | **SDNU 20241407** | **PZ403683** | **PZ403700** | **PZ403710** |
| ***A. tianshanica* 8** | **China** | **SDNU 20241393** | **PZ403682** | **PZ403699** | - |
| *A. tintickiana* 1 | USA | Leavitt 17-556 (BRY-c) | MH555411 | MH555427 | - |
| *A. tintickiana* 2 | USA | Leavitt 17-555 BRY-C | MH555410 | MH555426 | - |
| *A. turpanensis* 1 | China | Abbas 20179911 (XJU) | MK614802 | MK637538 | MK636515 |
| *A. turpanensis* 2 | China | Abbas 20140937 (XJU) | MK614803 | MK637537 | MK636514 |
| *A. turpanensis* 3 | China | Abbas 20169117 (XJU) | MK614804 | MK637536 | MK636513 |
| *A. umbilicata* | Sweden | Tibell 23532 (UPS L-136981) | LN810808 | LN810933 | LN810808 |
| *A. versicolor* 1 | Czech Republic | Malíček 12827 | MZ262720 | MZ262732 | - |
| *A. versicolor* 2 | China | SDNU 20240808 | PV355636 | PV355885 | PV355860 |
| *Glypholecia qinghaiensis* 1 | China | KUN-L 10-0241 | MZ330798 | OP749902 | - |
| *G. qinghaiensis* 2 | China | KUN-L 20-68255 | MZ330789 | OP749910 | - |
| *G. scabra* 1 | Norway | Westberg 08-232 (S) | LN810811 | LN810936 | - |
| *G. scabra* 2 | China | KUN-L 22-71500 | OP749917 | OP749900 | - |
| *Myriospora dilatata* 1 | Sweden | Nordin 5507 (UPS L-124304) | EU870660 | EU870712 | LN810871 |
| *M. dilatata* 2 | Sweden | Baloch SW116 (S F114109, holotype) | EU870656 | EU870708 | LN810872 |
| *M. smaragdula* 1 | Sweden | Ågren 384 (UPS L-098484) | EU870686 | EU870738 | LN810878 |
| *M. smaragdula* 2 | Sweden | Wedin 6620 (UPS) | EU870688 | EU870740 | LN810879 |
| *Pleopsidium chlorophanum* 1 | Sweden | Nordin 4439 (UPS L-076485) | EU870691 | EU870743 | LN810881 |
| *P. chlorophanum* 2 | Sweden | Nordin 6209 (UPS L-179248) | LN810813 | LN810938 | LN810813 |
| *P. flavum* | Austria | Obermayer 7790 (UPS L-105590) | AY853385 | AY853336 | AY853385 |
| *Pycnora sorophora* | Sweden | Hermansson 7903a (UPS L-111613) | FJ959357 | AY853338 | AY853387 |
| *Sarcogyne adscendens* 1 | California | Knudsen 6079 (H) | OP162367 | OP177753 | OP216685 |
| *S. adscendens* 2 | California | Dart 1230 (SBBG) | OK142749 | OK032134 | - |
| *S. algoviae* 1 | Norway | Westberg 08-276 (S F122564) | LN810849 | LN810976 | LN810849 |
| *S. algoviae* 2 | China | SDNU 20222129 | PP528132 | - | - |
| *S. basialba* 1 | USA | Leavitt S. 18139 (BYU) | PV873182 | PV883325 | PV883305 |
| *S. basialba* 2 | USA | Leavitt S. 24159 (BYU) | PV873189 | PV883318 | PV883297 |
| ***S. bayingolinensis* 1** | **China** | **SDNU 20240362** | PZ403686 | **PZ403703** | **PZ403713** |
| ***S. bayingolinensis* 2** | **China** | **SDNU 20240379** | PZ403687 | **PZ403704** | **PZ403714** |
| *S. belarusensis* 1 | Belarus | Golubkov 81 | OQ171090 | OQ184802 | OQ195853 |
| *S. belarusensis* 2 | Belarus | Golubkov 78 | OQ171091 | OQ184803 | OQ195854 |
| *S. clavus* 1 | Austria | Obermayer 09129 (GZU 49-2002) | LN810852 | LN810978 | LN810852 |
| *S. clavus* 2 | Sweden | Berglund SAR220 (S) | LN810853 | - | LN810853 |
| *S. coeruleonigrans* 1 | USA | Kocourková 10625 | OQ171104 | OQ184816 | OQ195866 |
| *S. coeruleonigrans* 2 | USA | Schultz 16242 | OQ171108 | OQ184820 | OQ195870 |
| *S. distinguenda* 1 | Sweden | Westberg 08–305 (S F120452) | LN810854 | LN810979 | LN810854 |
| *S. distinguenda* 2 | Norway | Haugan H3852 (O L17425) | LN810855 | LN810980 | LN810855 |
| *S. fallax* | Portugal | Zaca 2347 | MZ262722 | MZ262734 | MZ262744 |
| *S. hypophaea* 1 | Sweden | Westberg SAR198 (S) | LN810856 | LN810981 | - |
| *S. hypophaea* 2 | Finland | Pykälä 23561 (H) | LN810857 | LN810982 | - |
| *S. nimisii* | Italy | Nimis 35083 | OQ171133 | OQ184844 | OQ195892 |
| *S. nivea* 1 | Czech Republic | Malíček 12679 | OQ171100 | OQ184812 | OQ195863 |
| *S. nivea* 2 | Czech Republic | Malíček 14530 | OQ171085 | OQ184797 | OQ195848 |
| ***S. parasitica* 1** | **China** | **SDNU 20241125B** | **PZ403684** | **PZ403701** | **PZ403711** |
| ***S. parasitica* 2** | **China** | **SDNU 20241125A** | **PZ403685** | **PZ403702** | **PZ403712** |
| *S. platycarpoides* 1 | Italy | Nimis 34786 (TSB) | OQ171137 | OQ184847 | OQ195896 |
| *S. platycarpoides* 2 | Czech Republic | Malíček 2240 | MW715742 | MW715739 | MW715728 |
| *S. pruinosa* 1 | Czech Republic | Malíček 2560 (hb. Malíček) | OQ171097 | OQ184809 | OQ195860 |
| *S. pruinosa* 2 | Czech Republic | Malíček 6296 (hb. Malíček) | OQ171098 | OQ184810 | OQ195861 |
| ***S. yiliensis* 1** | **China** | **SDNU 20240065** | **PZ403688** | **PZ403705** | **PZ403715** |
| ***S. yiliensis* 2** | **China** | **SDNU 20240069** | **PZ403689** | **PZ403706** | **PZ403716** |
| ***S. yiliensis* 3** | **China** | **SDNU 20240093** | **PZ403690** | - | - |
| *Trimmatothelopsis rhizobola* 1 | Sweden | Westberg 2994 (LD) | EU870640 | EU870692 | LN810868 |
| *T. rhizobola* 2 | Sweden | Westberg 3099 (LD) | EU870641 | EU870693 | LN810869 |
| *T. terricola* 1 | USA | Knudsen 11216 & Sagar (S F256012) | LN810806 | LN810931 | LN810806 |
| *T. terricola* 2 | USA | Knudsen 11216 & Sagar (S F256013) | LN810807 | LN810932 | LN810807 |
| *Timdalia. intricata* 1 | Sweden | Westberg P114 (S) | LN810867 | LN810992 | LN810867 |
| *T. intricata* 2 | Sweden | Westberg SAR92 (LD) | LN810866 | LN810991 | LN810866 |
